# Novel closure technique using dual-action tissue clip with multi-bending scope for mucosal defects after gastric endoscopic submucosal dissection

**DOI:** 10.1055/a-2708-5736

**Published:** 2025-12-08

**Authors:** Hiroshi Ashizawa, Hiroyuki Ono

**Affiliations:** 1 Division of Endoscopy, Shizuoka Cancer Center, Shizuoka, Japan

Delayed bleeding occurs after gastric endoscopic submucosal dissection (ESD) in 4.7% of cases 1 , and mucosal defect closure reduces the risk 2 . However, existing clip-closure techniques often have difficulty achieving complete closure 3 . The dual-action tissue (DAT) clip (Micro-Tech Endoscopy, USA), a novel through-the-scope with a center-fixed column and two independent arms ( Fig. 1 **a, b** ), has been reported to be useful for closure after colorectal ESD 4 . We demonstrate a useful closure technique using a DAT clip after gastric ESD ( Video 1 ). A 71-year-old male underwent ESD for early gastric cancer of the lesser curvature of the antrum using a therapeutic gastroscope (GIF-H290T; Olympus, Japan). The lesion was resected en bloc with a 30-mm × 24-mm specimen. Air deflation was not achieved during defect closure when the DAT clip was inserted into the working channel. Therefore, switching to a multibending double-channel scope (GIF-2TQ260M; Olympus, Tokyo, Japan) allowed air deflation and reduced defect margin tension. The mucosal edges were approximated toward the opposite side of the defect after opening one arm and grasping the edge ( Fig. 2 **a, b** ). The other arm was used to grasp the opposite edge of the defect, and suturing was easily accomplished ( Fig. 2 **c** ). Four existing clips were placed for reinforcement, and complete closure was achieved within 3 min ( Fig. 2 **d** ). Complete closure was maintained on the day after ESD, and the patient was discharged on day 6. The delivery catheter of the DAT clip was 3 mm long, which is almost equivalent to the working channel of a therapeutic gastroscope. Therefore, air deflation and grasping of the opposite mucosal margin are challenging. By changing the multibending scope, sufficient air deflation was possible, and closure of the defect was easily achieved. Combining the DAT clip and multibending scope is an effective option for closure after gastric ESD.

**Fig. 1:**
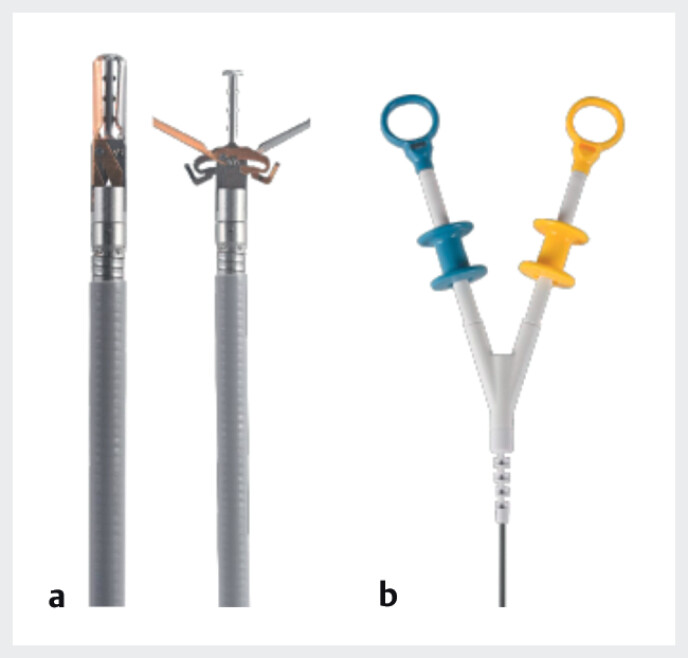
**a** The DAT clip is a three-arm TTS consisting of a central fixed column and two arms that operate independently. **b** The maximum opening width is 15 mm, and the angle is 60°. Two color-coded handles control the opening and closing of each arm. Source: Micro-Tech, Japan.

**Fig. 2:**
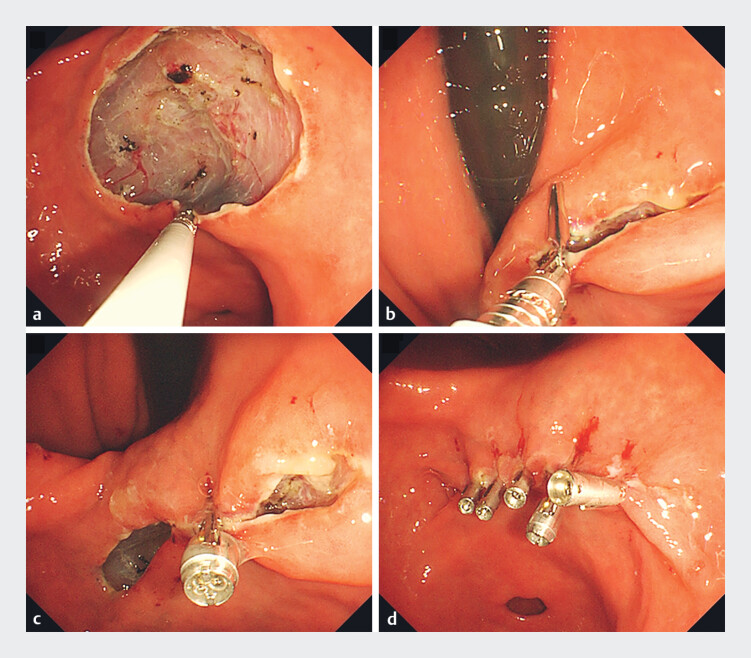
**a** Grasp the edge of the defect with 1 side of the dual-action tissue (DAT) clip (Micro-Tech Endoscopy, USA, Ann Arbor, Mich, USA). **b** After air deflating the stomach, relieving tension of the defect margin, and reducing the mucosal defect, the grasped mucosa is approximated toward the opposite edge, and the second arm of the DAT clip is used to grasp the opposite edge. **c** Successfully closed the center of the mucosal defect. **d** Complete mucosal closure was achieved in 3 min using four additional clips (Sure Clip, Micro-tech, USA).

Video showing the dual-action tissue (DAT) clip being used to close a mucosal defect after gastric ESD. Combining the DAT clip and multibending scope makes closure easier. Source for DAT clip: Micro-Tech, Japan.Video 1

Endoscopy_UCTN_Code_TTT_1AO_2AO

## Notes

Endoscopy E-Videos https://eref.thieme.de/e-videos
*E-Videos* is an open access online section of the journal *Endoscopy* , reporting on interesting cases and new techniques in gastroenterological endoscopy. All papers include a high-quality video and are published with a Creative Commons CC-BY license. Endoscopy E-Videos qualify for HINARI discounts and waivers and eligibility is automatically checked during the submission process. We grant 100% waivers to articles whose corresponding authors are based in Group A countries and 50% waivers to those who are based in Group B countries as classified by Research4Life (see: https://www.research4life.org/access/eligibility/ ). This section has its own submission website at https://mc.manuscriptcentral.com/e-videos .
